# Onset of Weight Gain and Health Concerns for Men: Findings from the TAP Programme

**DOI:** 10.3390/ijerph19010579

**Published:** 2022-01-05

**Authors:** Mark Cortnage, Andy Pringle

**Affiliations:** 1 School Allied and Public Health, Anglia Ruskin University, Young Street, Cambridge CB1 2LZ, UK; 2 Human Sciences Research Centre, University of Derby, Kedleston Road, Derby DE22 1GB, UK; a.pringle@derby.ac.uk

**Keywords:** obesity, football, weight gain, sedentary, weight stigmatization, physical activity, self-esteem, men, self-objectification

## Abstract

With shown reticence by men to engage with dietary interventions for weight loss, investigations that provide detail on men’s perceptions for the causes of weight gain and subsequent concerns over health and image are important. Such discoveries have potential to make a valuable contribution to male gendered programme design aimed at tackling weight gain and promoting good health. Connecting to men to health using their hobbies and interests, this study deployed semi-structured interviews of eight male participants (age > 35 years) enrolled on The Alpha Programme (TAP). TAP is a 12-week football and weight management intervention delivered in local community venues. Results captured men’s lived experiences and feelings of being overweight, their attempts at dietary modification, health and causes of weight gain. Results signify externalized attribution for weight gain, entrenched habitual intake practices, despondency related to weight stigmatization, self-objectification and low self-worth. Moreover, this study outlines the processes for capturing this information using a male friendly approach and setting. Outcomes have potential for shaping bespoke men’s weight management and health improvement interventions in the future.

## 1. Introduction

Feelings of being overweight and obese have limited research coverage. Recognising the low engagement with health services, men may not consider incentives such as dieting and health improvement as strong motivators to tackle weight, whereas alternative incentives may be more enticing, namely opportunities to improve performance and effectiveness [1]. Complementing insight into male perceived barriers to weight loss, by understanding perceptions of weight and the contribution of dietary related behaviours would help shape behavioural approaches accordingly.

Traditional masculine norms negatively ascribe help seeking behaviour with weakness, loss of control and autonomy [2,3]. For men to seek help risks stigmatisation [4] that if internalised can lead to further feelings of negativity towards counselling [3]. Although younger men are more inclined to engage in ‘performative acts’ such as risk taking, violence and excessive drinking [5], older men, perceived to be more risk adverse [6,7] portray a cautious appreciation of risk where age related decline in strength, fitness and sexual prowess manifest into efforts to halt said decline [8]. Yet, despite this perception, older male, health positive practice is not replicated in the UK data which underlines a persistent increase over the last 28 years of male overweight/obesity, rising from 58% of the total UK male population in 1993 to 68% in 2019 [9]. Across the age range 45 to 74 years 79% of the UK male population are either overweight or obese. UK males have increased mortality from avoidable disease than women at 150.2 deaths per 1000 (*n* = 3896) and 97.4 per 1000 (*n* = 2705) in 2019, respectively. Since 2011, the greatest slowdown in mortality improvement for men is ischaemic heart disease [10].

Stigmatisation has been associated with externalisation of identity, outlining self-objectification, e.g., ugly, horrible [11], often facilitating a degradation in emotional wellbeing [12,13]. In men Lozano et al. (2016) [14] found that weight stigma undermines men’s sense of self-concept and men’s masculine values and becomes a social threat—real or imagined—that entails negative psychosocial outcomes, preventing men’s participation in social activities, including weight loss. Habitual unhealthy eating practices are capable of weakening self-efficacy to the point of undermining the effectiveness of commonly recommended dietary methods, e.g., planning and self-monitoring [15]. By exposing the thoughts of respondents as to the cause(s) of their weight gain, we can tailor more effective intervention.

A male study sample (*n* = 35) ages 35 to 64 years participated in a 91 week, optimised gendered football and nutrition programme named The Alpha Programme (TAP) which targeted weight loss as an empirically evaluated outcome. Qualitative feedback obtained from interviews was used to compare outcomes and evaluate the success of the intervention and identify key implementation considerations. These lived experiences are presented through themes identified related to health, weight, and diet.

For this programme, development of the Community of Practice (COP) [16] is facilitated through shared common interest, namely football. Knowledge exchange, shared experiences and personal development are particularly important to men who commonly display tendencies for social isolation, degraded mental health state and subsequent reduced health service engagement [17]. When coupled with stigmatisation in relation to weight we portray a demographic that may engage with health incentives through a conducive environment populated by likeminded individuals with a firm, yet empathetic Transformation Leadership [18].

## 2. Materials and Methods

### 2.1. Intervention Context

The Alpha Programme (TAP) was conceived in 2013 based on both empirical and research outlining the reluctance of men to access health services and engage with effective weight management incentives [19,20,21] with the aim of offering an innovative male gendered alternative to weight loss and management. TAP commenced on the 25 July 2015. Initial interviews were conducted during this week. The programme ran for a period of 91 weeks, considerably longer than the initial plan of 12 weeks. TAP achieved significant weight loss and maintenance at 91 weeks.

### 2.2. Ethical Consideration

Ethical principles 1 to 6 of the Economic and Social Research Council [22] were adhered to in all methods used in this study. The protocol outline was explained to the participants during the induction interview and participants were free to ask for clarification at any time. Stage 1 Ethical approval was sought from the Anglia Ruskin University Faculty Research Ethics Panel on a single occasion. Approval was accepted from the date 23rd June 2015 for three years. Reference Number: 15/026.

This research followed the best ethical guiding principles, specifically related to weight management programmes at the time. Research ethics in practice were aligned with Have al., (2013) “Ethical framework for the prevention of overweight and obesity” [23].

### 2.3. Instrumentation

The study utilised a convenience sampling method, a form of non-probability or non-random sampling where the sample were required to meet certain criteria, such as accessibility, proximity to the research and convenience [24], to recruit as many men as possible onto the programme. 

For recruitment, leaflets were left at the reception of the football ground where the sessions were to be held. Leaflets were also divided between the researcher and volunteer coach who distributed to friends and acquaintances. Potential participants were able to contact the researcher directly via the contact details on the leaflet, register for attendance in person through the researcher or coach or register their interest at the football venue.

All participants completed an induction session consisting of a presentation prior to commencement of training. The presentation was followed by the completion of documents; two Participant Consent Forms (PCF) and one Participant Information Sheet (PIS). Alongside written details outlined in the PIS, further information about the study was presented to all participants during induction. The opportunity to ask (and address) questions continued throughout the session. Upon acceptance of the details included on the consent form and research protocol, recruits were asked to sign two copies of the PCF, with one copy handed back to the participant and the other retained by the researcher. All participants were provided with the PIF and encouraged to retain it.

Interviews are regularly used to investigate participants. In this study, interviews were voice recorded to help improve transcription accuracy. Each interview was digitally recorded using an Olympus WS-832 digital voice recorder. A further backup digital recorder, the Olympus DS-40 was used simultaneously in case of failure. Interviews were recorded in MP3 format and uploaded onto an online secure drive after recording. Recordings were transcribed against each question asked, with respondents identified by initials to protect anonymity. A quality control check was conducted, playing back the interviews whilst reading the transcripts to ensure completeness.

For the interviews with participants the semi structured approach used allowed the researcher to ask open ended questions and helped avoid imposing opinions and assumptions onto the interviewee [25]. Participants had ‘free reign’ to respond how they wished. To avoid any leading or cohesion, the researcher used brief questions with very little interruption once the participant was responding. Interviews ranged between 45 to 55 min in length, with one participant completing the interview in under 30 min despite the best attempts of the researcher to encourage expansion on the answers provided. Interview sessions were conducted Pre-programme phase 1 (initial 12-week programme). Eight participants were interviewed (Table 1). All participants were approached by Mark Cortnage when attending TAP training sessions and asked if they would like to be interviewed.

#### Analysis

Thematic content analysis was used for qualitative analysis of interview data, allowing for a rich, complex account of the interviews to be used to identify themes with detailed meanings [26]. The method provides the means to explore experiences and feelings in separate specific accounts (in relation to questions). As outlined by Braun and Clarke [27], the ability of this method to reflect the reality of experiences shared and to ‘unpick the surface of reality’ justifies its choice for use here. Responses generated by the semi-structured interviews held at baseline and at the end of the intervention were coded using a thematic approach. 

Coding was conducted using Braun and Clarke [27] ‘Phases of Analysis’, a six stage process that helped the researcher conceptualise the process of thematic analysis) (Table 2). Though recognised more as a guide than a set of rules, the researcher adhered closely to the guidance set out.

The thematic analysis was conducted manually to enable the researcher to remain close to the data, gaining a thorough understanding of the interviews. Different coloured pens were used to identify and represent themes, providing a visual representation which facilitated a quick glance method of identifying theme development across large swathes of data. On completion of the interviews, the researcher had formed an idea of the types of themes that were present in the data and spent time uncovering as much detail on those as possible when conducting the analysis. Once a theme began to emerge, even vague references such as single words were highlighted, enabling analyses to recognise their contribution to the overall picture.

## 3. Analysis

The lived experiences of men who participated in the programme are presented through themes identified related to health, football and diet. Four themes were uncovered through thematic content analysis of the interview transcripts, and these are further developed in the discussion (Table 3).

This section provides interview findings focusing on the male relationships with food and diet. The men openly discussed the period into which they began to gain weight and suggestions as to the cause, their feelings as to what it is like to be overweight. We can see how perceptions of lifestyle restricts good eating practices and relate to how the men attributed weight gain to two externalised factors; family and employment, providing insight suggesting that they deemed the condition to be out of their control [28]. Analysis outlined strong habitual and routine practices and lack of activity that were viewed by the men as central to weight management. Furthermore, two men had engaged heavily in cyclic dietary behaviours with the period of weight regain significantly shorter than the period of weight loss. Health was seen as of some concern by the men but there was little inclination before joining the programme to address levels of risk. Low self-esteem was prevalent, with negative portrayals of body image manifesting as self-objectification further accompanied by accounts of external sigmatisation.

### 3.1. Attribution of Weight Gain

It was made evident that lifestyle events decreased the opportunity to exercise. Conversations suggested three reasons for weight gain: lifestyle (including family commitments), occupation supporting a decrease in activity and poor, habitually led food choices. Often, the termination of exercise and the entrance into an alternative, often family centred lifestyle happened simultaneously, and the men would often view them as being incompatible by suggesting that a lifestyle event prevented exercise rather than integrating the two.

Mr R:… *I suppose since when the kids arrived. I’ve always been quite active, always played football, always done something and then the kids came along, that stopped so before you know it you’re not younger I was eating the same sort of stuff because you’re exercising it’s going off… burning it off, but stopping the exercise. It’s because there’s less time you’re eating more convenient*.

Sobal, Rauschenbach and Frongillo [29] 10-year longitudinal study suggested single men after marriage had a mean weigh gain of 7.4 kg over that period, for men who were already married at baseline, weight increase was 5.8 kg. The 16 year study conducted by Mata et al., [30] suggest significant weight gain in married men of up to up to 0.833 kg/m^2^ over that period. Weight increase was determined despite controlling for weight-related behaviours such as age, children, employment suggesting weight gain would be difficult to attribute to a single influence or event rather a whole-lifestyle approach. With around a third of adults spending their life in work, levels of occupational activity can have a significant impact on total daily energy expenditure [31]. The 2016 study by Chin, Nam and Lee [32] posit that managerial occupations, with less activity related tasks were significantly associated with lower aerobic exercise engagement outside of the workplace and suggest how influence on one aspect of lifestyle can impact on another.

Mr E: I suppose… *I mean, there’s more pressure at work now because I’ve got more of a high… more of a managerial role. So, it has… where work roles changed so there’s more responsibility and more time there. So, there’s less sort of I suppose flexibility in when you eat and stuff like that but I need something quick and get on with it*.

Mr C: *I have been putting on weight for years, Mark. I stopped playing football about the age of 35–40ish but because I run my own business, well, I can manage my work when I play football, training and stuff like that, and I wasn’t eating as much, but when I stopped playing football and started to settle into life and with having my own business and taking clients out, as you do when you have clients, you take them out for a meal, or a drink and the easier life in relation to social events was like the big thing, so I started to put the weight on*.

A slew of research have suggested that the rise in obesity prevalence coincides increased levels of physical inactivity, wider food choice and unstructured eating behaviours, etc. [33,34,35]. Recognising strong associations between sedentary behaviour and obesity [36,37], the TAP men provided rare insight into how accessibility to convenience foods coupled with sedentary behaviour contributed to their gradual weight gain.

Mr R: *Then you probably have a cup of tea with some biscuits although you didn’t need it but they’re there and you go down that slippery slope and possibly if there was a beer, you’d go and have a beer or something*.

Mr E: Crisps. *If they’re not in the house, it doesn’t bother me but if they are in the house, I’ll have to have a packet*.

Mr C: *I think there may be something about starchy stuff that makes you sort of addicted, but I know for a fact that chocolate and crisps are one of my biggest fall-downs. I will be sitting there or might be driving somewhere, and I put a Mars Bar in my mouth rather than an orange or something like that*.

Recognition by participants that they do not need the food suggests that restraint was felt to be beyond them. Despite best intentions, the desire to eat tempting food, whether consciously or not overrides individuals intended behaviours. Regular consumption of treats promotes a relationship between sensory signals and the feeling of satiety that the food presents.

Mr E: *You know, I’ve just sat a bit in the evening you know, you’re comfortable, you are relaxed, chilled watching the telly or something like that… my biggest downfall which I’m concentrating on at the moment is I like to pick in the evening. It wouldn’t necessarily bother me if I don’t eat during the day*.

Mr E provided insight to support research hypothesis that distracted eating has been shown to increase energy consumption [38,39]. The repetitive nature of this eating pattern highlights habitually unconscious consumption practices. One method which has some efficacy in arresting distracted eating is to improve attentiveness when eating and may be a beneficial approach under such circumstances [38,40].

I: *So, what foods do you know you shouldn’t eat but find hard to resist?*

Mr S: *Probably crisps… probably savouries more than sweets you know. You know, if somebody said to me in the evening, sitting down do you want a bar of chocolate or a tub of Pringles, I’ll go for Pringles*.

This emphasises the potential for convenience to overwrite intentions. Over consumption of regular meals was not a focus of blame, rather confectionary items were. Evening inactivity is unlikely not be the primary reason for daily positive energy, rather a combination of Total Daily Energy Expenditure exceeded through a combination of high fat and high sugar consumption and sedentary behaviours. Research outlines how sedentary aspects can influence poor food choices [41] with suggestions that a combination of reward cues such as stress, boredom and habit are responsible [42,43] and may outline the reasons as to why these men engage in these snacking activities after work.

### 3.2. Concerns over Health and Weight

The age in which weight gain were thought to have emerged were varied and attributed to external factors and indicated that participants had little motivation to address them once recognised. Three participants mentioned health without the topic being directly addressed and outlined a possible maturing of perceptions in some which contrasts with suggestions that men have little concern for their health or take measures to seek help [44,45,46]. However, all three respondents had prior health concerns, which positively influenced their perceptions and motivations to address them. Research does suggest that men, post health ‘scare’ may be more receptive to intervention [47,48]. Mr C worked in IT and largely sedentary throughout the day, participating in little or no exercise at other times. Mr E worked in the Information Technology (IT) field as a manager. His working day was of a sedentary nature and similarly to Mr C, he had developed underlying health conditions in relation to his weight and yet had performed little in the way of preventive action to address these.

I: *What concerns do you have about being overweight?*

Mr C: *Well, my biggest concern is that after putting on a lot of weight I had a heart attack about 15 years ago, so I don’t want to go back into that situation again. I’ve had no problem since then, but a lot of the problem was due to diabetes. I want to see my days out; I want to be single again and enjoy life instead of carrying on being fat*.

Mr E: *I need to lose weight to help with my blood pressure and get me off the tablets because I hate taking tablets at the best of times and I have to take a stupid amount now. What is it, four tablets I take a day… and if I lose weight there’s no reason why I then have to take… you know, or the doses come down you know?*

Mr B worked in a routine classified occupation as a delivery driver and had had a battery of tests performed a year before joining the programme. Most of his daily routine was driving, taking cargo from one destination to another. He performed very little exercise prior to joining the programme. His cholesterol, blood pressure and resting heart rate were normal (supported through the medication mentioned), and he had lost and regained five and a half stone, and which had culminated into concerns about diabetes due to this rapid weight gain. Despite his health concerns, Mr B. attended intermittently (citing that work commitments prevented his regular attendance).

Mr B: *Yeah, same with cholesterol as well, that was it, cholesterol test they did. That was right in the middle, that was 5, fine. Before I lost my 5½ stone last year, I put it all back on in less than a year… You could say that my blood sugar level now may be a lot higher because I put it on so quickly, so again. So, I’m aware and conscious that there’s health issues, if you know what I mean*.

The remaining men only discussed health when the topic was pursued by the researcher and answers focused more on levels of fitness and age rather than the risk of disease associated with being overweight or obese. Other than for two men, preventative approaches to ill-health seemed of little concern, suggesting a disconnect between the benefits of exercise, good diet and reduced health risk. Mr R worked as a car salesman at the time of interview and had a mainly sedentary occupation and though he would take the occasional walk at the weekends, he remained sedentary in his spare time and recognised that his limited fitness motivated him to enroll on the programme. Health was not a motivator for enrolment and only mentioned slight concern of health risk when questioned. Prior to joining the programme he appeared to have done very little to address these concerns.

I: *Which concerns do you have about being overweight such as health for instance?*

Mr R: *Yeah, it is like short of breath probably if you are overweight and if you, do you know even because I don’t do any exercise in the winter. So, if you have to find anything out like a couple of months we were in London with my son and we had to go up the escalator in the underground and I was knackered. The same escalator as like eight years ago when I used to work there. I used to fly up and down and now it is like… that’s it. It was absolutely a killer. I was short of breath*.

Mr R: *I suppose I’ve never really had any health concerns, but I suppose as I’m getting older and you see everybody else that you know… it’s slowly started to drift in the back of your mind your bodies sort of telling you you’ve got to start doing something*.

Although action was taken by some men to address health concerns, the majority appeared indifferent. Considering their age, reticence may be attributed to embarrassment and/or fear to express themselves [49] or attributed to male gendered performances relating to health risk [50].

### 3.3. Prior Dietary Attempts

The following section displays attempts by the researcher to uncover efforts by the men to redress increased weight in particularly, their level of engagement with diets and of the level of success. Recognising that TAP was primarily a nutrition programme, with an aim to help men to lose and maintain weight suggested that long term maintenance of weight had not been achieved prior to joining. However, information related to prior dietary attempts would help address the lack of research regarding men and diets.

Mr B: *Huh, I’ve done all sorts of different sorts of diets, Huh, um last year I did a really good healthy eating, I wouldn’t call it diet. But it’s smaller amounts of food, more regularly, uh, maintaining your blood sugar level so you don’t get cravings, you don’t feel hungry and that worked for the time I did it. But then I put all the weight back on after … Yeah, 5½ stone, uh in 18 weeks*.

I: *Gosh, so you rebounded big time mate*.

Mr B: *Yeah, you know down, personal training, uh badminton lessons 5 times a week and then yeah, just put it all back on. You need to maintain that so you can say eat one day whatever you want and then six days. And then when it’s you on your own you go—ah—I’ll make it 2 then I’ll make it 3 and then it just slips back.*

Mr B: *I did Slimming World probably 6 years ago*.

I: *How did that go?*

Mr B: *Lost 5 stone on that and put all that back on… there’s a 5 stone mark here isn’t there, mean mentally when I get to that mark I stop*.

Evidence highlights that men are averse to diets which are embodied as a ‘purely female’ pursuit [51], with exercise preferred as a means to control weight [52,53]. Men are more inclined to favour individualised, structured and fact-based dietary approaches [54] and once engaged in weight loss, are shown to lose weight more quickly than women [55]. Mr B suggests a rebound will be expected at the 5 stone mark and may hinder further help seeking although with Mr B weight loss achievement was through several days per week of activity and may explain how football is a motivator for participation [56]. Although 18 weeks of activity and weight loss is admirable for Mr B, we are presented with an individual who from a sedentary state, with little exercise conditioning participated in activity daily. Successful weight maintenance strategies outlined by Ramage et al., [57] are associated with decreased energy intake, higher quality food choice, increased activity and behavioural control around food. Recounting two rather successful weight loss attempts followed by weight gain for Mr B suggests that the plethora of facilitators for maintenance had not been accommodated or integrated into lifestyle to such an extent that the behavioural and physical adaptations required to support maintenance become routine. If withdrawal from the sport was forced upon him through, e.g., injury then re-engagement may be difficult unless acclimatisation had been achieved.

With earlier evidence suggesting [6,7] that older men are risk averse and more inclined to see help around health, the dietary patterns of weight loss accompanied by continued high-risk behaviour inducing weight gain, suggest otherwise and highlight a cyclic approach to dieting.

Mr C.: *So, I started to put the weight on but I sort of ‘yo-yo’ dieted. I would go on a diet and lose about 3 stones and then I would put it all back on again*.

Rapid and large gains in weight observed in some men over short periods of time reflect high-risk behaviours seen within this group. Rapid regains are associated with reduced resting energy expenditure (REE) [58] and weight cyclers can develop poorer hormonal and metabolic profiles [59]. The participants most affected by persistent weight cycling were unable to show restraint or recognise harm. Although this approach is not solely related to men, evidence does suggest that this is a masculinised health risk behaviour [60,61]. Research on male weight cycling is limited and more commonly addressed in sporting related research [62,63,64] and yet we can see (above) that for some men losing weight is referred to as an achievement and a natural approach to weight loss rather than maintenance. Investigations in male weight cycling should be considered specifically for men outside of sporting circles and their perception of weight maintenance.

### 3.4. Feelings about Being Overweight/Obese

It was felt that an exploration of what being overweight or obese felt to the men. We see how the men refer to having excess weight in derogatory terms, such as ‘slug’, ‘crap’ portraying self-objectification manifesting as feelings of worthlessness, shame [65] which individually or in combination, support a perpetuation of negative food and activity related behaviours [66,67]. Comments also bring into focus the relationship between weight status and mental health state.

I: *How does it feel to be overweight?*

Mr E: *I don’t like it*.

I: *What sort of feelings does it give you…?*

Mr E: *I feel pretty crap… you know, I sort of like get up in the morning and whatever and walked past a mirror and I think, look at that gut… but I know I should do something about it. It makes you feel pretty down really to be totally honest*.

Mr T: *It just makes me feel like a slug. It just makes you feel a bit depressed or whatever*.

Mr R: *Sluggish, tired, yeah. Low self-esteem*.

Below, we are further greeted by uncertainty over weight status and brings into clarity a lack of awareness of risk. We are also presented with a lack of knowledge between exercise adaptation, ageing and body size with lower-than-expected performance attributed to weight. A common occurrence with new starters on the programmes was an expectation by participants that they would be able to perform on the pitch to a similar level than before they had retired from playing. This was alleviated through a periodisation approach that supported a gradual increase in intensity over the 12-week period although enthusiasm to perform at a level consistent with their youth was rarely blunted and had to be continuously monitored to reduce injury.

Mr F: *I don’t like it because I’ve never been overweight…… but it’s more… I mean, I’m overweight I wouldn’t say… I suppose technically I’m obese I would imagine from the way they do it now. … but I don’t feel huge when I’m walking around all day or anything like that…… but I notice it when I play five-a-side and my legs get tired like they never did before and I’m sure that must be as much weight as age*.

Mr E: *Because I don’t feel big… because of the size of my chest and stuff like that, I sort of hide behind that, well I’m a big lad, you know? Chest, I’ve got a big chest and I’ve got some boobs now but… I’ve got a huge chest you know, and I look at it and I just went, oh! When I saw a picture, I went core blimey, you are a big lad but my arms are thin, my legs are thin, I just look like a barrel. Yeah, it’s that and I think if I could lose that…*

The previous example highlight the potential for mental health to be affected as a result, with feelings of depression openly mentioned and in contrast to the expected stoic response expected [68]. The reciprocal association between obesity and depression has been repeatedly shown [69,70,71]. Information as to whether obesity causes depression or visa-versa remains uncertain [69] and by asking such a question, this research could have further contributed to that debate.

Improvements in wellbeing for men can be achieved through increases in physical activity, greater peer support and social integration, etc., and presented as a mechanism to alleviate depressive symptoms [72,73]. However, as with most interventions targeting men to date, these are reactive approaches, whereas pro-active intervention, prior to development of symptoms may be a far more effective strategy.

In this study, men reported the absence of health professionals enquiring into how they felt about their obesity. When men are contemplating making lifestyle changes that can positively impact on their weight, the lack of intervention can be considered a missed opportunity. Guidance highlights that physical activity performed regularly can contribute to management of obesity [74]. Healthcare professionals have been identified as being key when promoting health enhancing behaviours such as physical activity [75]. In preparing to intervene, it is also important that healthcare professionals are aware of the behaviours that men demonstrated to deflect their discomfort of being overweight such as being stoic, macho or humorous, as well as the detrimental impact that negative feelings of being obese have on mental wellbeing. Further training and education could be helpful to enhance the preparedness of the Health Care Practitioner to routinely ask men about their feelings about being overweight. This can establish a platform to intervene, especially when men have presented at healthcare settings under their volition.

In alignment with research consensus that for men in particular, recognition of excess body weight risk does not necessarily manifest into a modifying behaviour [76,77] it was recognised that upon recruitment, none of the men in TAP were considered well placed to self-motivate and may have felt vulnerable and open to potential ridicule when joining the programme. Their decision to engage, however, appears to have contributed to levels of confidence and through participation with like-minded individuals in similar circumstances, appears to have helped foster the solidarity that was targeted within the intervention design. Alongside on-programme peer support, peer networks to aid recruitment have been shown as a viable strategy to improve uptake [53].

Stigmatisation is a common accompaniment to being overweight/obese and again associated with negative food related behaviours [78,79]. In addition to self-objectification, perceptions of image extend to other’s perception of the subject that even the closest of relationships may be strained by, with a belief that the individual is viewed with disgust. Furthermore, we see (below) how shopping for suitable clothing induces frustration, stress inducing and further perpetuate feelings of shame.

Mr C: *I have been yo-yoing up and down for years Mark. It’s been a problem. I think you get comfortable—my daughter nags me to death, she’s healthy and she’s quite fit and my partner is too and my kids are all quite slim so I am like the ‘blob’ of the family and so to a degree it tends to get a bit depressing after a while when you hear that and sometimes when you hear it so often, you think I can’t be arsed. But I’ve got to this stage now where I’m sitting there thinking that I’m going to meetings and I looking at mirrors and I’m wearing a shirt and tie and I’d like to be wearing a suit but I can’t get into that suit because I can’t get one in my size so I think I’m trying to focus the mind now and trying to get back to a simple way. Ideally, in my case, I’d love to lose 10 stone*.

*Personally, at times I feel depressed, well maybe not depressed. I feel down, what gets to me the worst is buying clothes and you go into a shop and see a really nice suit and you know they won’t have it in my size and a size 54 chest you know is getting quite ridiculous. It’s got to stop*.

*I do feel embarrassed to be quite honest because the jokes come thick and fast and you laugh with them as part of your make up and you stick by it—it’s not killing anybody is it—it hates fat people*.

Mr C provides insight into how approaches to discuss his weight appear infrequent. There is no mention of medical personal enquiring over his feelings, outlining risk or signposting for possible remediation. Furthermore, suggestions portray that he has experienced indirect responses that may attempt to make light of his weight, in this instance alluding to his eligibility for compensation, whether due to comorbidity or mortality is not made clear. Lastly, we are reminded of his discomfort at having to enrol on a programme that addresses men of a certain weight and may further contribute to a reluctance by men to participate. However, programmes, in this instance, conjuring feelings of resignation of weight status and related comorbidities may be the first step in rehabilitation and posit programmes that align behavioural support on developing acceptance of weight status as an alternative approach.

Mr C: *You are the first person who has ever asked me that. How do you feel? Yes, I feel shit. I would love not to be part of this programme. I mean, this programme is good and I like it because I like playing football*.

### 3.5. Final Remarks

Low perception of health status risk is recognised in obese adults [80] with underestimation of weight reporting as less binge eating and eating disorder symptomology. Such behaviour, although at first may appear positive, the lack of awareness of true weight status may lead to an exacerbation of risk. 

Evidence from interviews and research highlights attribution where treatment response is influenced by individual perceptions of the condition and its causes [81]. For the participants, attributions were more often externalised, placing the cause of their predicament out of their control. This was is in contrast with research, where internalised causes are more typically dominant, e.g., overeating to obesity [81,82]. External, less ‘controllable’ behaviours are likely to increase despondency and demotivation, presenting as repeated behaviours contributing to the condition [83] for instance, weight cycling behaviours which has been shown to have a significant relationship with all-cause mortality [84]. 

Patterns of onset weight gain, exercise, self-image and weight loss were contextualised within the lifestyle behaviours of the men were identified. Personal characteristics and experiences shared provided variable levels of emotional insight, highlighting how low self-esteem and lifestyle constraints, e.g., employment accompanied continued high-risk behaviours rather than intervention: facilitating persistent, yet gradual weight gain despite awareness as to how said behaviours negatively influence health. Self-objectification presented a recurring image of poor self-worth and highlighted nuanced self-esteem. Although discourse focused on body image, detrimental comments associating body size and agility (slug) highlighted particularly male gendered connotations with performance on the pitch and highlighted the benefits of providing a programme focusing on ability and skills development to men rather than match-play alone when referencing self-esteem. A similar intervention strategy had been taken with women to some success [85]. 

Improvements in food knowledge have been shown to improve self-efficacy by enabling the individual to make informed choices [86,87] and social support derived through regular engagement in sport is also proven to be effective in developing self-esteem [53] both of which were utilised with this programme. However, research suggests that participation in exercise for improvements in image may exacerbate feelings of self-objectification, disordered eating and compromised body esteem [88,89] and should be used with caution when used for motivational development. Theories around masculinity however suggest that engagement in exercise for performance and aesthetic improvement is an inducement for male participation [90,91]. The approach used here concentrated on health improvement through a combined diet and fitness approach since although aesthetic improvements and on-pitch performance and physical performance improved as a result, the protocol was designed to ensure these were not overtly addressed to avoid arresting self-esteem development. 

Results indicate that men are vulnerable to cyclic dietary behaviours. Eating during comfort breaks were suggested as having strong associations with weight gain and yet despite the awareness of these actions and health risk, attempts to redress were infrequent and lacked commitment.

Two life course events were attributed to their predicament, namely family and employment with discussions telegraphing a sense of resignation and that weight gain was an inevitable consequence of these life choices. Discourse on their weight status were often tinged with despondency and portrayed an erosion of self-esteem, and yet, despite a keen awareness of both their mental state and the inevitable increase in health risk over the life course were their behaviours to continue, action to induce sustainable positive change were not made evident, with attempts to redress being infrequent and non-committal.

The men placed significant emphasis on activity as being a primary regulator of weight and highlighted poor awareness of the multiple negative influences on weight status other than comfort eating. In this regard, and as provided through TAP, men would benefit from incentives that have an educational component that helps develop a holistic understanding of weight and lifestyle that aligns more keenly to their gendered perceptions.

Sharing these findings with services focused on weight loss in men such as commercial and statutory health providers could be helpful in establishing weight loss goals that were not only realistic and sustainable but also seen as credible and as such inclusive of those men who aspired to change their weight loss status and improve their health profiles.

## Figures and Tables

**Table 1:** Participant demographics and phase(s) when interviewed.

Name	Age	BMI (kg/m^2^)
Mr C	40	49.9
Mr E	43	39.6
Mr B	50	44.7
Mr R	43	38.9
Mr T	35	32.1
Mr C	51	28.2
Mr F	58	29.1
Mr S	54	32.4

**Table 2:** Phases of analysis used to develop themes.

Phase	Process Description
1	Transcribing of the data. Generation of ideas
2	Generation of codes. Process conducted across the entire data set as opposed to individual interviews.
3	Searching for themes.
4	Reviewing the themes. A review to ensure that the themes align to the codes.
5	Defining and naming of themes.
6	Producing a report based on the themes.

**Table 3:** TAP pre-programme interview themes.

TAP Programme: Recognised Interview Themes
Attribution of weight gain
Concerns over health and weight
Prior dietary attempts
Feelings about being overweight/obese

## Data Availability

The data presented in this study are available on request from the corresponding author. The data are not publicly available due to ethical restriction.
